# Ultrasound a New Paradigm in Regional Anesthesia and Pain Management

**DOI:** 10.5812/aapm.13363

**Published:** 2013-09-01

**Authors:** Poupak Rahimzadeh, Seyed Hamid Reza Faiz

**Affiliations:** 1 Department of Anesthesiology, Hazrat Rasul Medical complex, Iran University of Medical Sciences (IUMS), Tehran, Iran

**Keywords:** Anesthesia, Conduction, Pain Management, Ultrasound

To perform nerve block accurately; imaging to increase safety and maintaining the whole integrity is essential. In the early twentieth century, paresthesia was used to detect the correct location of the nerve but was gradually replaced by more secure methods such as nerve locators and nerve stimulators. During recent years, extremely attention has been focused on nerve stimulation in the regional anesthesia and pain management field but it has a passion for attending all the experts in this field of practice. Perhaps it was due to different individuals and different diseases which cause the nervous stimulation limits. Diseases such as diabetic associated neuropathy can make the nerve stimulator almost worthless. Other neuropathic conditions such as toxic neuropathies, demyelinating conditions, even peripheral vascular and advanced age can fade the response to the nerve stimulator. Placement of the electrode with poor return and inconsistent contact in the active electrode can further take dawn the issues. 

During the 1980, the idea of using ultrasound as a safe guide in the field of regional anesthesia was proposed. This equipment has the capability of real time dynamic evaluation of the tissues while is showing live image. On the other hand, lack of radiologic exposure danger, comfort ability and low cost of its usage makes this device an acceptable option for the majority of anesthesiologists and pain physicians. The machine itself is more affordable than a fluoroscope, computed tomography scan, or magnetic resonance imaging machine. These characteristic and continued improvements in image quality and resolution have expanded the use of ultrasound in many areas in medicine beyond traditional diagnostic imaging applications. Nowadays by coming ultrasound machines in the more anesthesia wards a new revolution has been started in the field of regional anesthesia and pain management; while as an increasing willingness toward performing different ultrasound guided nerve blocks has grown up. Actually, Ultrasound imaging has become a popular technique to perform regional anesthesia and pain procedures (1-3). The ability to view and track needles during performing ultrasound nerve blocks is one of the indicators that is considered proficient in working with ultrasound. Other indicators include familiarity in operation with the machine, properly interprets and optimizes the images. There are some general advices while doing ultrasound scan. One should have a practiced approach for each nerve using landmarks and borders that he/she can follow during performing the procedure. Different approaches are suggested for each type of regional block on the literatures. Usually more than one approach is suggested for a single block. This is reasonable due to the fact that when a single approach will not work on a given patient’s body habitus especially for a deeper nerve; the operator can choose another alternative approach. Vascular structures are usually easily differentiated from other targets, in the case of existence of the color Doppler mode by the ultrasound machine; it can be used to detect flow.

Tendons and ligaments can look like nerves on ultrasound. Both of them in general appearance are much more echodense from more angles than the nerves, but this similarity is usually hardly detectable. Both tendons and ligaments tend to be located below the level of nerves, closer to the bone. Slowly moving the limb may produce noticeable movement in a tendon on ultrasound screen while a nerve would not move much. When scanning transverse for nerves, slightly changing the angle of the ultrasound probe along the long or short axis in the most cases which result in a better quality of the nerve image (4-6). There are some other advices while doing procedures: One should organize the procedure so that the attention can be focused on the target shown on the ultrasound screen during placement of the needle and the initial injection of the local anesthetic mixture. Also, the operator always should not to inject all the local anesthetic mixture via a single needle approach and should reposit the needle at least twice under ultrasound observation and perform second or further injection.

However, this method has its specific limitations. Limits of high resolution in deep tissues, especially in obese subjects, artifacts caused by bony structures. Lack of enough expertise and how to scan a picture and change in picture by shifting the position of the probe - as the interpretation completely depends on the experience of the person using the probe-, next to sonovisible equipment costs and times to learn this skill that leads to the limitations of its usage, but by considering all the advantages of this method, it seems worthy to do (5, 7-10). Sonoanatomy is a new field which has been introduced to increase the success rate of ultrasound guided procedures (11, 12). In Iran there have been a growing number of centers which are using ultrasound for regional anesthesia and pain procedures but there are still a distance between Iran and developed countries in this field, fortunately a new enthusiasm with lots of attention in this field has been grown up and it is assumed that by creation of this great attention; this gap will be reduced quietly soon. Iranian society of regional anesthesia and pain medicine (ISRAPM) is an independent forum which has many members between Iranian anesthetists and pain physicians. Society is made up of 5 years before and has done many actions to further spread this knowledge to other professionals interested in using ultrasound for regional anesthesia and pain procedures in the form of workshops which has been hold at least twice per year by the scientific team of the society. The society in fact has an important mission in this way and has tried to improve the ultrasound knowledge and has continued its attempt. Hope in the near future by creation of more acceptance on the efficacy of ultrasound by more anesthesia and pain departments for using this method to perform all nerve blocks and pain procedures, an increasing in safety and succession rate occurs both for the patients and doctors.
